# Supplementary material for: Hemostasis System and Plasminogen Activity in Retrochorial Hematoma in the First Trimester of Pregnancy
Source: Biomedicines. 2022 Sep 14;10(9):2284. doi: 10.3390/biomedicines10092284 (PMC9496394; doi:10.3390/biomedicines10092284)
Supplement: Supplementary file 1 [file biomedicines-10-02284-s001.zip › biomedicines-1829343-supplementary.pdf]

Supplementary Files

Table S1. The pattern of the distribution of hemostasis parameters in the Shapiro-Wilk W–test.

Index	Normal pregnancy, N=57	Retrochorial hematoma, N=16
Prothrombin time (PT), s	W=0.84649, **p=0.00000**	W=0.93511, p=0.29331
Activated partial thrombin time (aPTT), s	W=0.93174, **p=0.00317**	W=.96886, p=.81984
Thrombin time (TT), s	W=0.94843, **p=0.01670**	W=0.92278, p=0.18698
Fibrinogen, g/l	W=0.97871, p=0.41072	W=0.91309, p=0.13054
Soluble Fibrin Monomeric Complexes (SFMC), mg%	W=0.65547, **p=0.00000**	W=0.66552, **p=0.00007**
D-dimer, ng/ml	W= 0.6301, **p= 0.00000**	W=0.68269, **p=0.00011**
Plasminogen (PG) activity, %	W=0.95700, **p=0.04122**	W=0.86859, **p=0.02588**
Plasminogen concentration, mcg/ml	W=0.71368, **p=0.00000**	W=0.78619, **p=0.00180**
IgG-plasminogen, units	W=0.75293, **p=0.00000**	W=0.61536, **p=0.00002**
IgM-plasminogen, units	W=0.93112, **p=0.00299**	W=0.67346, **p=0.00009**
IgA-plasminogen, units	W=0.95438, **p=0.03119**	W=0.94682, p=0.44101
PG/IgM-PG activity	W=0.89123, **p=0.00010**	W=0.92566, p=0.20796
gestational age	W=0.90858, **p=0.00039**	W=0.94141, p=0.36670

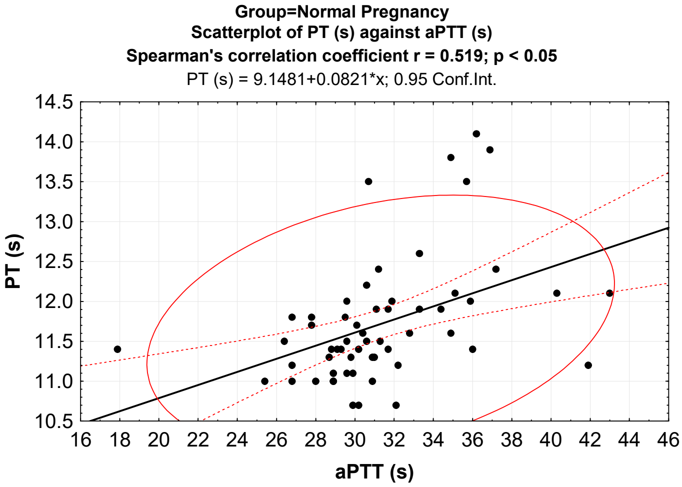

(A1)

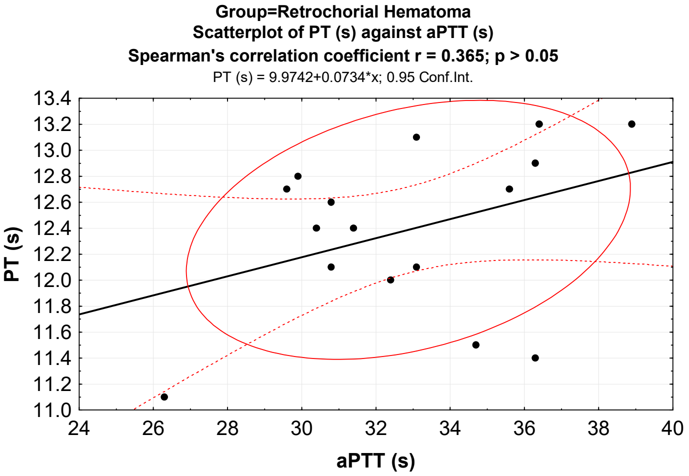

(A2)

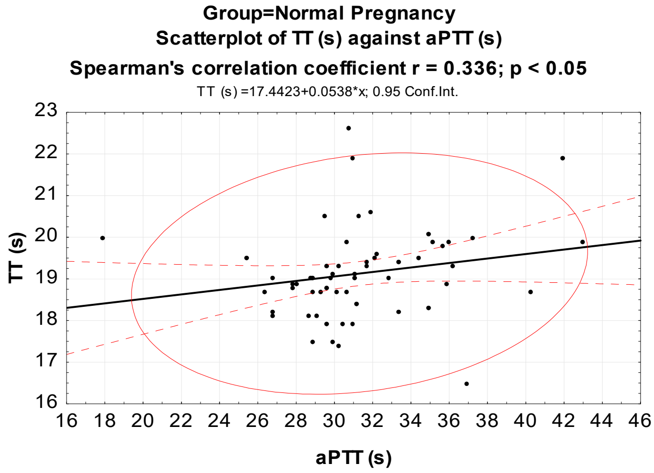

(B1)

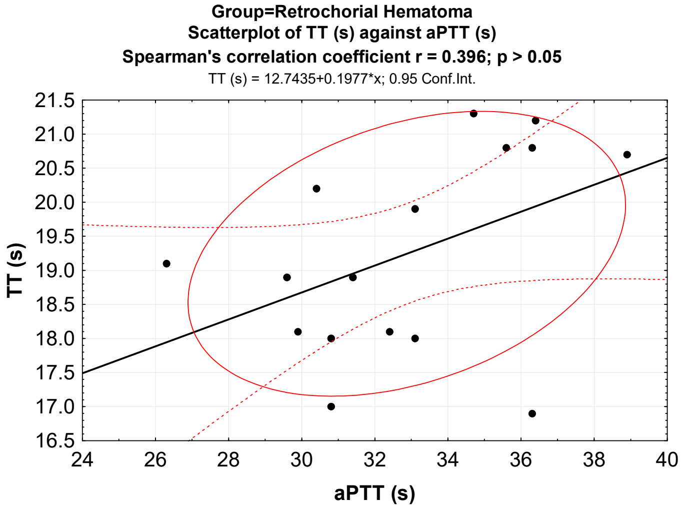

(B2)

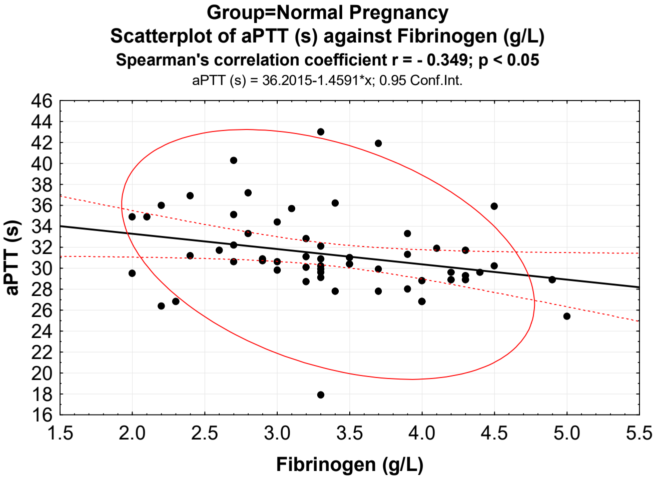

(C1)

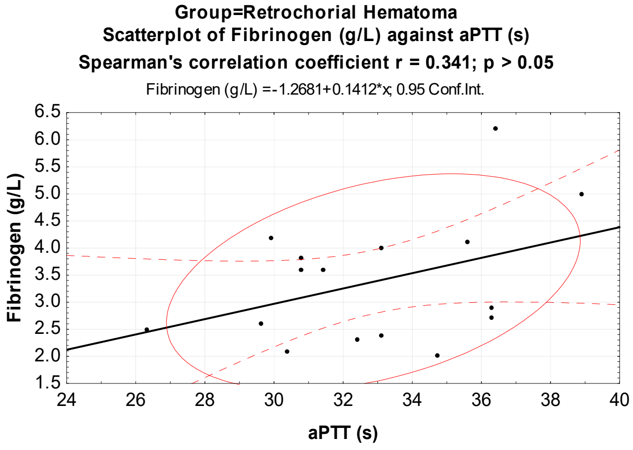

(C2)

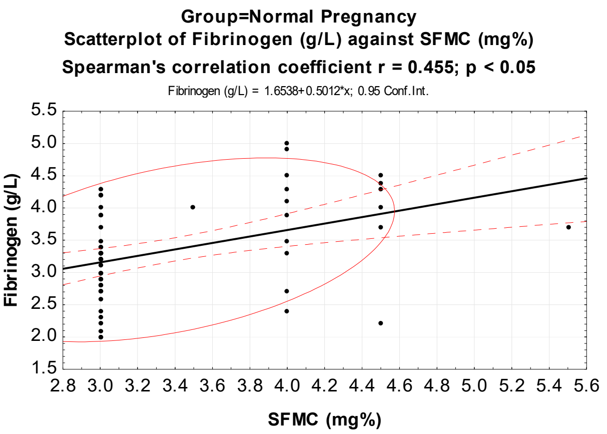

(D1)

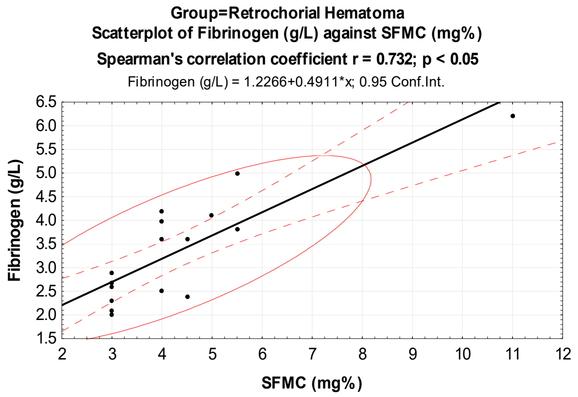

(D2)

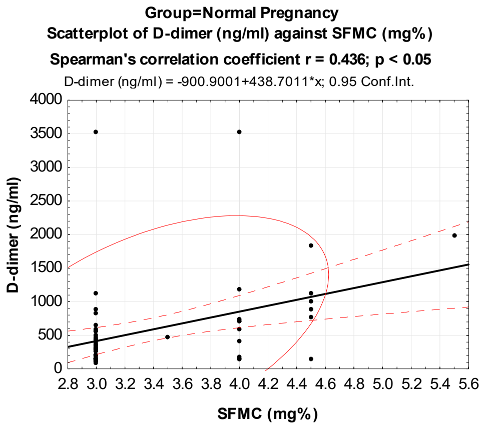

(E1)

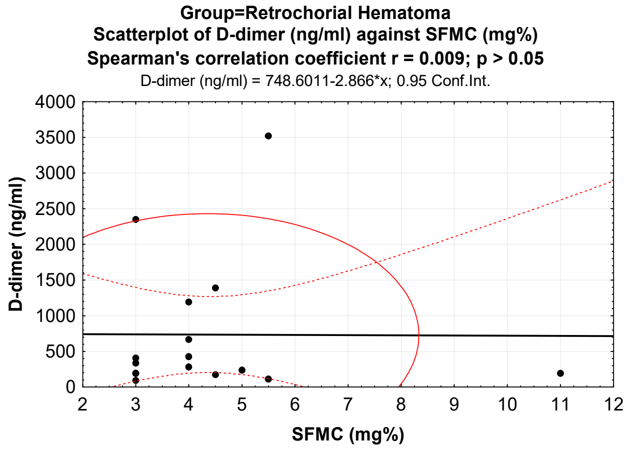

(E2)

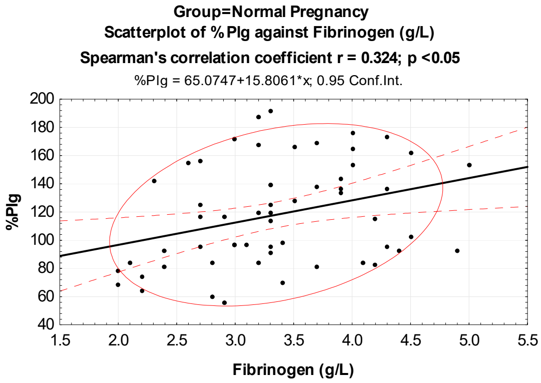

(F1)

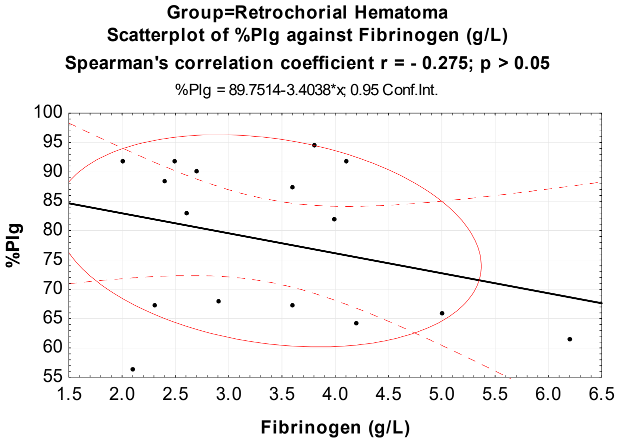

(F2)

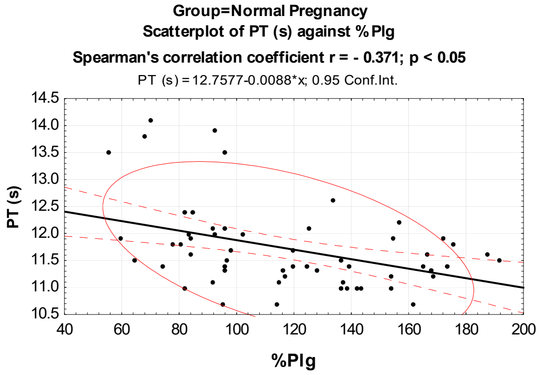

(G1)

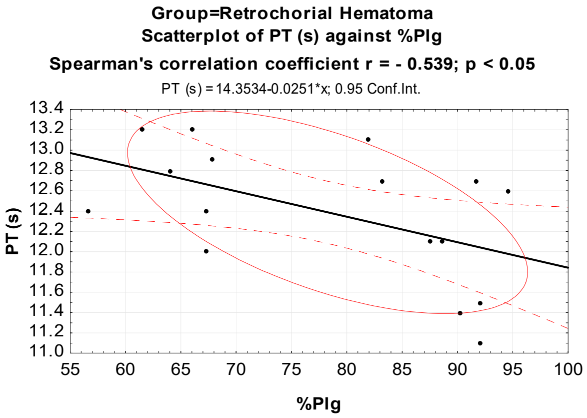

(G2)

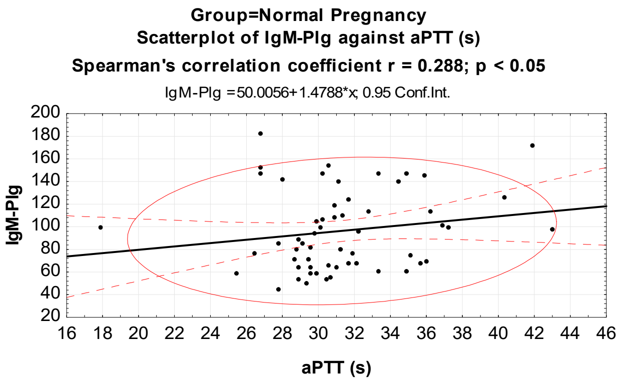

(H1)

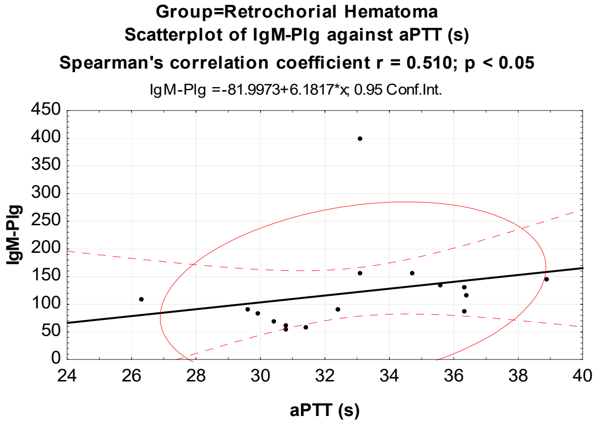

(H2)

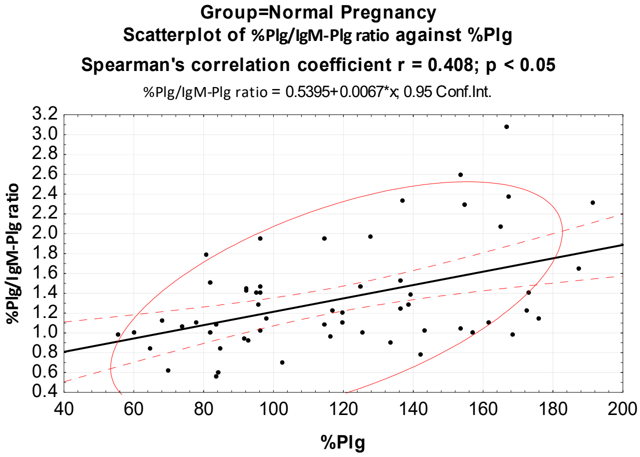

(I1)

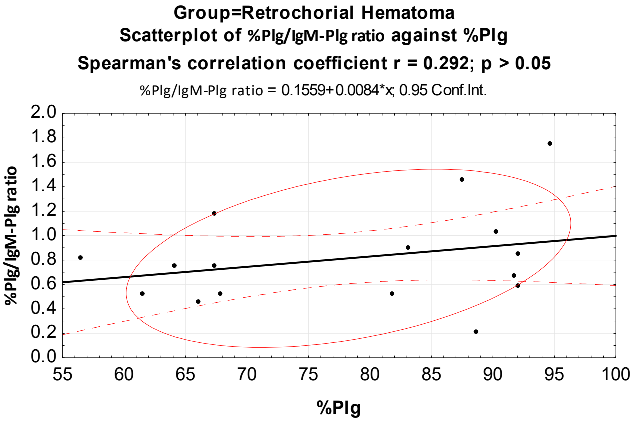

(I2)

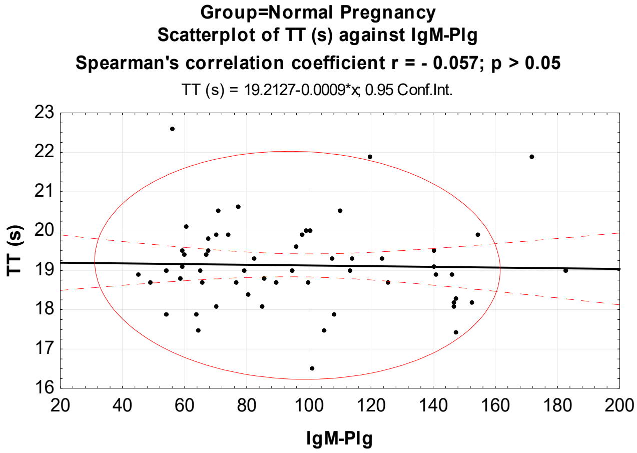

(J1)

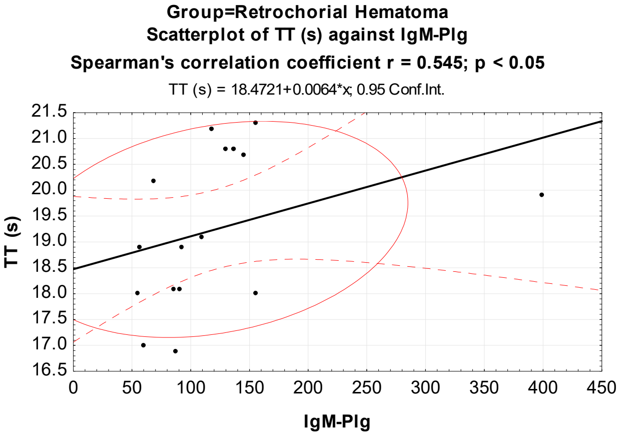

(J2)

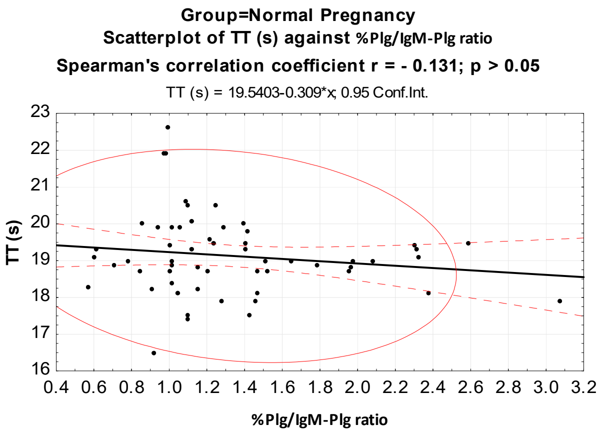

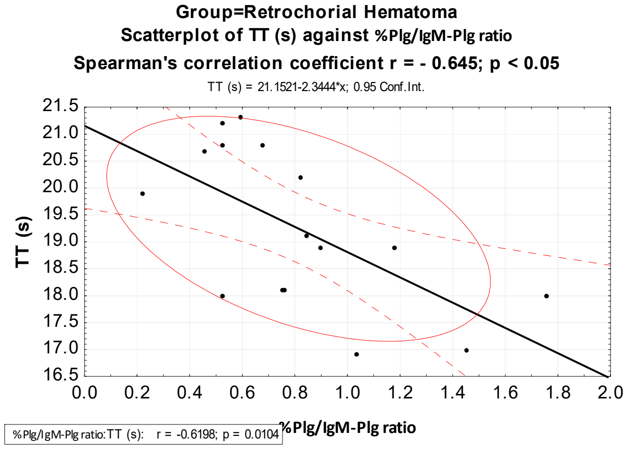

(K1)

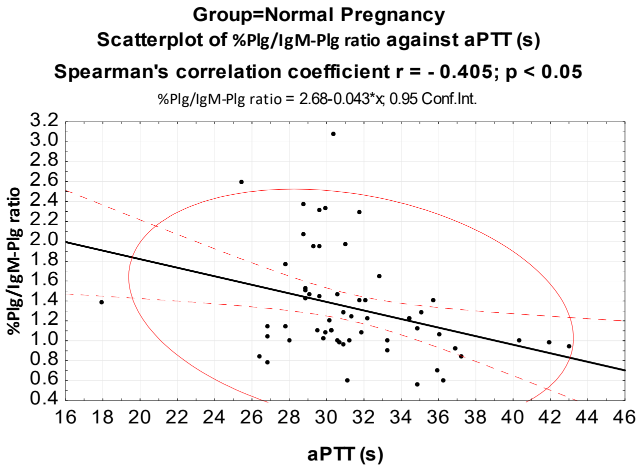

(K2)

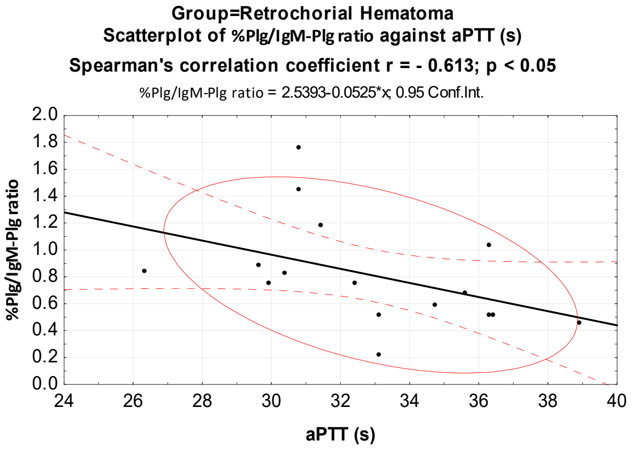

(L1)

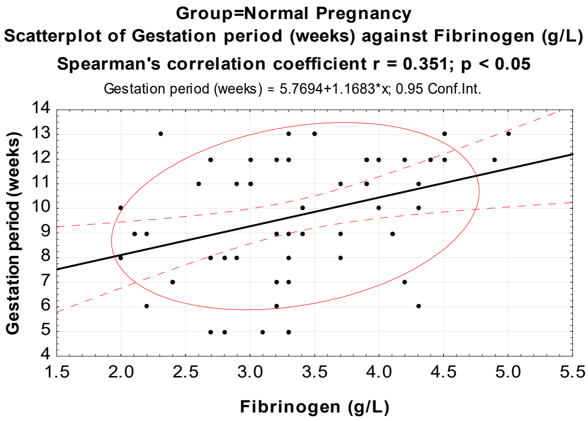

(L2)

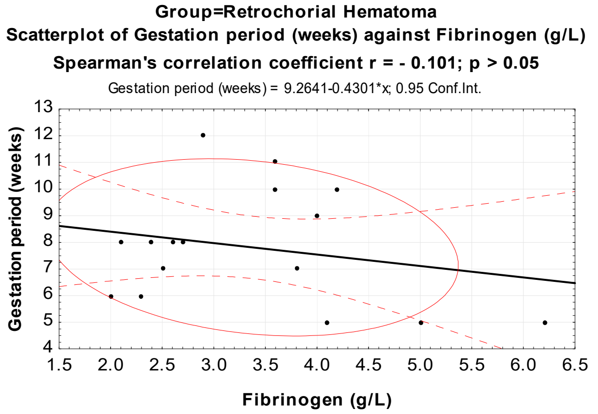

(M1)

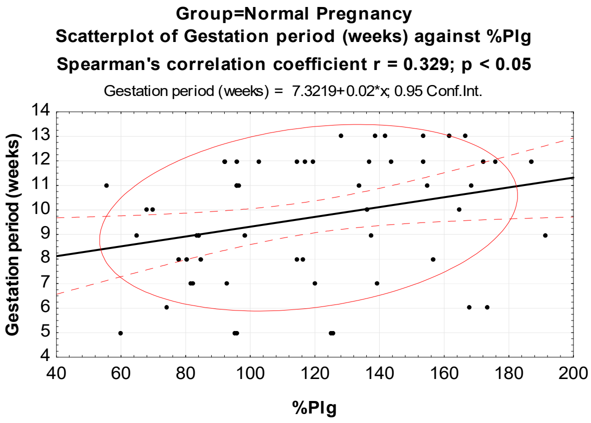

(M2)

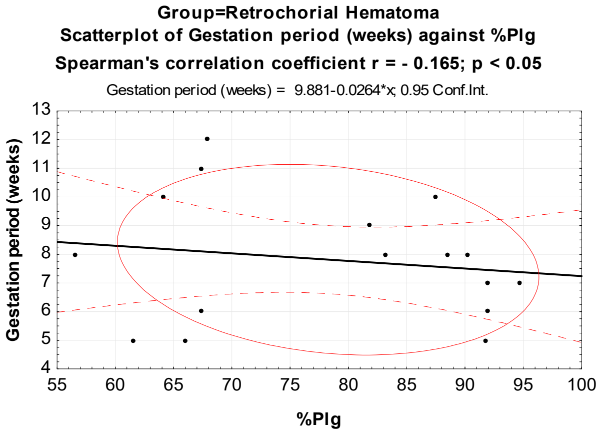

(N1)

(N2)

Figure S1. Scatterplots of hemostatic parameters in normal pregnancy (n=56) and with retrochorial hematoma (n=16).
